# Inari large-bore mechanical thrombectomy in intermediate-high risk submassive PE patients: Case series and literature review

**DOI:** 10.21542/gcsp.2022.8

**Published:** 2022-06-30

**Authors:** Mohammad F. Mathbout, Hussam Al Hennawi, Anwar Khedr, Katrina Bidwell, Thomas M. Todoran

**Affiliations:** 1 Medical University of South Carolina, Department of Cardiology, Charleston, South Carolina, USA; 2 Department of Internal Medicine, Jefferson Abington Hospital, Abington, PA, USA; 3 Department of Critical Care Medicine, Mayo Clinic Health System, Mankato, MN, USA

## Abstract

Pulmonary embolism (PE) is one of the most common causes of cardiovascular (CV) mortality worldwide. Owing to the associated morbidity and mortality with other treatment modalities, including systemic thrombolysis, a discernible change in the era of acute pulmonary embolism management has been reported. Catheter-directed thrombectomy using the FlowTriever system (Inari Medical; Irvine, CA, USA) was shown to reduce endpoints of interest in patients with acute intermediate-high risk PE and was associated with rapid hemodynamic improvement. In this report, we describe our experience with three cases of patients presenting with submassive PE, whereby immediate pulmonary artery pressure improvement was evident in all cases after successful mechanical thrombectomy. Our experience supports the use of FlowTriever mechanical thrombectomy for the treatment of submassive PE in clinical practice, with a call for further research to establish associated benefits.

## Introduction

Followed by acute myocardial infarction and stroke, pulmonary embolism (PE) constitutes the third leading cause of cardiovascular mortality and is a significant cause of in-hospital mortality with 60,000–100,000 deaths per year in the United States^1^. Intermediate-risk (submassive) PE presents in up to 40% of patients characterized by hemodynamic stability with concomitant right ventricular (RV) strain or cardiac injury, or both^2^. Risk stratification of patients with PE is of significant importance, as patients presenting with intermediate-risk PE have up to 21% mortality at three months. Novel interventions have been introduced owing to renewed interest in optimizing PE management, especially among patients presenting with hemodynamic instability. Anticoagulation remains the cornerstone of treatment for PE^3^.

Treatment options can be grouped into three distinct categories: systemic thrombolysis, catheter-directed interventions, and surgical thrombectomy. Randomized clinical trials have demonstrated significant benefits for intermediate-and high-risk PE managed with systemic thrombolysis^4^. Nevertheless, this benefit has been countervailed by an elevated risk of major bleeding and intracranial hemorrhage, restricting its use in clinical practice^4,5^. Among other modalities, catheter-directed thrombolysis involves catheter-mediated direct injection of thrombolytic agents into the affected pulmonary artery. The role of FlowTriever embolectomy and Indigo thrombectomy system has been investigated in this cohort of patients and was recently approved by the US Food and Drug Administration for clinical use^6^. Nevertheless, there is a lack of solid evidence to evaluate possible mortality benefits in patients undergoing catheter-based thrombectomy using the FlowTriever system. Therefore, the application of such modality remains subjective to patients according to their risk of bleeding, location of thrombus, and operator skills until well-defined guidelines direct a unified clinical application. In this case series, we present three cases in which FlowTriver thrombectomy was used to successfully manage submassive PE.

### Case 1

**Figure 1.**
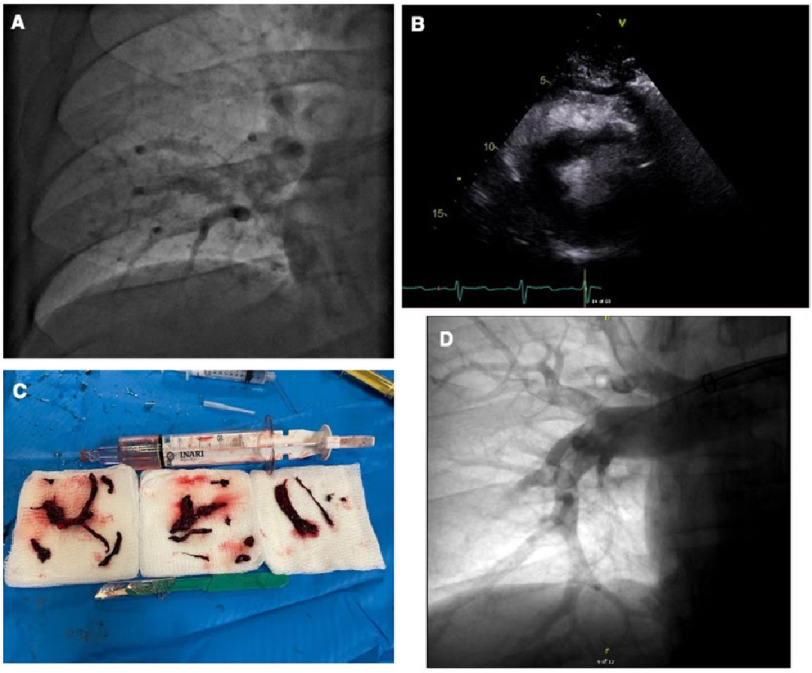
Pulmonary CT angiogram showing a large thrombus burden in the distal right main PA before thrombectomy (A). Right ventricular clot burden shown by transthoracic echocardiogram (B). The transthoracic echocardiogram shows large mobile thrombi in the right ventricle. Thrombus removed from the right PA with the Inari device. D: Final pulmonary angiogram after thrombectomy (C).

A 37-year-old male patient presented to the emergency department of an outside hospital complaining of squeezing chest pain for two days. The patient had a medical history of hypertension and an episode of PE that occurred five years ago. However, he did not undergo any hematologic workup at that time. The patient was taken off anticoagulants two years after the first PE. He worked as a forklift driver and reported sitting for long periods during his work. During the interview, he experienced an episode of syncope. He underwent a CT pulmonary angiography that revealed bilateral PE with filling defects in the right and left main pulmonary arteries and saddle embolus at the bifurcation (Figure 1A), with evidence of right heart strain confirmed by a transthoracic echocardiogram (Figure 1B). His troponin and pro-BNP levels were elevated.

The patient was admitted to the hospital for further management. In the cardiac catheterization lab, he underwent FlowTriever thrombectomy. On the procedure table, the patient’s systolic blood pressure was 174/95 mmHg, heart rate was 105 bpm, and O_2_ saturation was 92% on 4 L/min O_2_. Pulmonary artery catheterization was achieved through the right femoral vein using an 8 French introducer sheath. A pigtail catheter was placed in the main PA, and angiography was performed. A near-occlusive thrombus was observed in the distal right PA. Hemodynamic measurements showed a pulmonary artery (PA) pressure of 80/40 mmHg (mPAP:50 mmHg), a right atrium (RA) pressure of 12 mmHg, and a PA O_2_ saturation of 62.8%. A 26 French Gore Dryseal sheath (Gore, Flagstaff, AZ, USA), was introduced to advance the FlowTriever system (Inari Medical Inc., Irvine, CA, USA), which consists of a trackable 95 cm long, 24 French aspiration catheter used to retrieve the clot mechanically.

Thrombectomy of the right PA was successfully performed, with minimal blood loss and no complications. The 24 French FlowTriever catheter was advanced to the distal right PA and multiple aspirations were performed with significant visible thrombus extracted (Figure 1C). After the procedure, systolic blood pressure normalized to 124/74 mmHg, heart rate decreased to 94, and O_2_ saturation was 98% on room air. Repeat PA pressure immediately decreased to 55/18 mmHg (mPAP: 30 mmHg). Repeat CT angiography showed no clot in the distal right PA, with near-complete restoration of pulmonary blood flow (Figure 1D).

The patient tolerated the procedure well and was switched from heparin drip to Eliquis 5 mg twice daily. He was counseled to restart his home medications, and was discharged the following day with a hematology referral to investigate a possible underlying hypercoagulability disorder.

### Case 2

**Figure 2.**
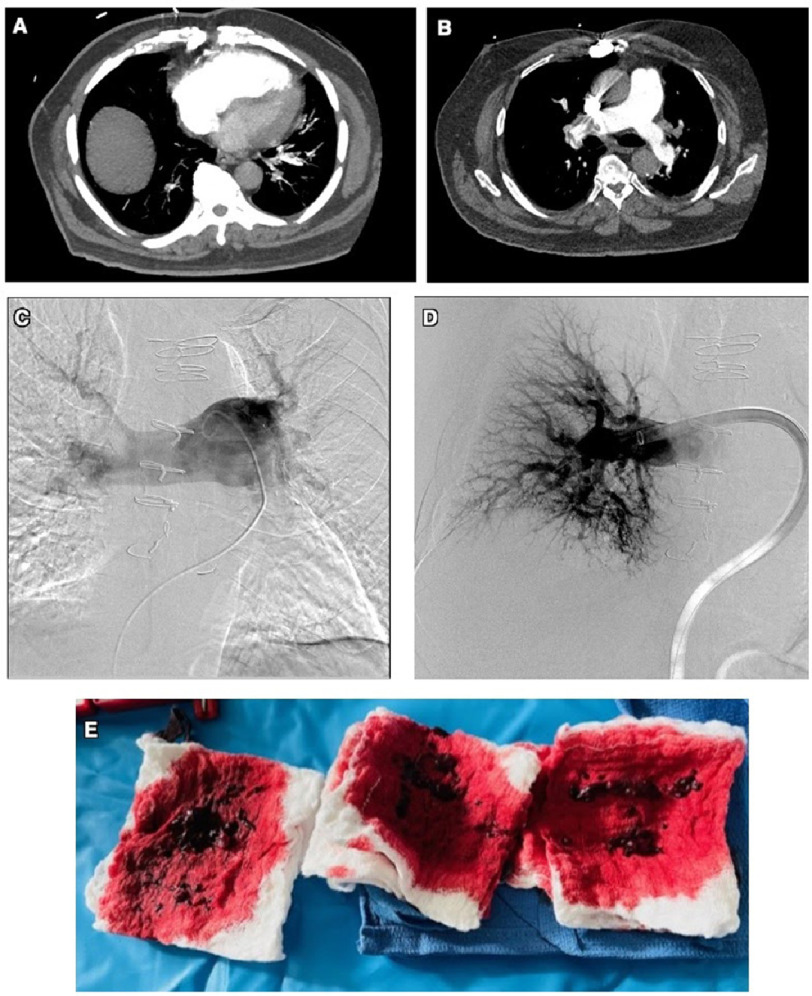
Computed tomography angiogram of the chest showing extensive bilateral pulmonary embolism with near occlusive thrombus in the distal right pulmonary artery and non-occlusive thrombus in the left lower lobe and upper lobe pulmonary arteries (A). Axial cardiac view showing an enlarged right ventricle with a right ventricle/left ventricle ratio of 1.68:1, indicating right heart strain (B). Angiogram showing a near-occlusive thrombus in the distal right pulmonary artery (C). Post aspiration angiogram showing restored flow (D). A large amount of clot was extracted, with no haemodynamic instability (E).

A 79-year-old male patient was transferred to our hospital for advanced management of bilateral submissive PE associated with borderline hemodynamics. The patient had a medical history of coronary artery disease post-3-vessel coronary artery bypass graft, hypertension, hyperlipidemia, and obstructive sleep apnea. He also complained of two years of progressive dyspnea on excretion. However, the patient had not been on home oxygen therapy. On admission, the patient underwent chest CT with contrast using PE protocol (CTPE), which showed a near-occlusive thrombus in the distal right pulmonary artery (Figure 2A) associated with flattening and leftward bowing of the interventricular septum with a right ventricle/left ventricle (RV/LV) ratio nearly 1.68:1 consistent with right heart strain. There was also a non-occlusive thrombus in the left lower lobe and upper lobe pulmonary arteries (Figure 2B).

The patient was transferred to the cardiac catheterization lab. Pulmonary artery catheterization was performed via the right femoral vein using an 8 French introducer sheath, which demonstrated a PA pressure of 60/20 mmHg (mPAP: 32 mmHg), PA O_2_ saturation of 67.5%, RA pressure of 10 mmHg, right ventricular (RV) pressure of 60/10 mmHg, and CO/CI 3.56/1.75. A pigtail catheter was advanced to the right PA, and angiography was performed, which showed a near-occlusive thrombus in the distal right PA (Figure 2C). A 26 French Gore Dryseal sheath was introduced to accommodate the FlowTriever system.

The distal right PA was selectively engaged with the 24 French FlowTriever catheter, and aspiration thrombectomy was successfully performed after multiple aspirations (Figure 2D). Left-sided thrombectomy was not performed because of decreased burden of the thrombus on angiography. On the table, repeat PA pressure improved to 50/10 mmHg (mPAP:30 mmHg) A pulmonary angiogram showed near-complete restoration of pulmonary blood flow to the right and left sides (Figure 2E). Following the procedure, the patient was initially treated with 6 L of O_2_ and was discharged on room air with an O_2_ saturation >90%.

### Case 3

A 48-year-old female with a medical history of hypertension presented to an outside hospital complaining of progressive chest pain, shortness of breath, and weakness for two days. She had a recent prolonged hospitalization course because of COVID-19 pneumonia complicated by acute respiratory distress syndrome. She underwent intubation and venovenous extracorporeal membrane oxygenation (ECMO) followed by pulmonary rehabilitation. Initial blood pressure was 130/82 mmHg, heart rate was 116 bpm, and O_2_ saturation was 97% on a 2L nasal cannula. Admission CTPE revealed a large bilateral saddle PE. Admission troponin was 0.1 ng/ml, and BNP was 697 pg/ml. She was started on intravenous heparin and was sent to the MUSC for further intervention. On arrival to ICU, the patient remained mildly tachycardic with a heart rate of 110 bpm. Her blood pressure was 108/83 mmHg, and O_2_ saturation was 100% on a 3L nasal cannula. The Pulmonary Embolism Response Team was called on arrival, and the patient was transferred to the cardiac catheterization lab for further management.

**Figure 3.**
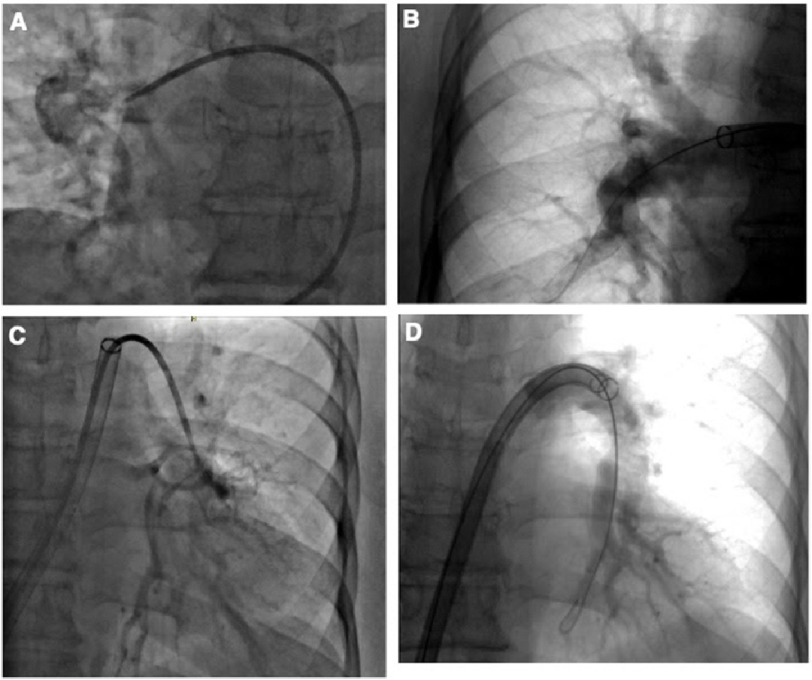
Right pulmonary artery (RPA) and left pulmonary artery (LPA) angiogram. Angiogram showing large thrombus at the distal right pulmonary artery (A). Post-thrombectomy angiogram showing patency of the right interlobar artery and right lobe artery (B). An angiogram showing a large thrombus in the distal left pulmonary artery (C). Post-thrombectomy of the left lower lobe anterobasal and posterobasal branches was successfully performed (D).

Pulmonary artery catheterization was performed using an 8 French introducer sheath through the right femoral vein, demonstrating a PA pressure of 50/24 mmHg (mPAP: 30 mmHg), RA pressure of 6 mmHg, RV pressure of 50/6 mmHg, PA O_2_ saturation of 51.7, and CO/CI of 3.54/1.62. A pigtail catheter was advanced into the right pulmonary artery, and angiography was performed. A large thrombus was observed in the distal right pulmonary artery (Figure 3A). A 24 French Inari FlowTriever catheter was advanced to the level of the right interlobar artery and then to the level of the right lobe artery, and aspiration thrombectomy was successfully performed through both arteries (Figure 3B).

The catheter was repositioned into the left pulmonary artery and angiography was performed. A large thrombus was also observed in the distal left pulmonary artery (Figure 3C). The curved 22 French catheter was advanced to the level of the left lower lobe anterobasal and posterobasal branches, and aspiration thrombectomy was successfully performed (Figure 3D). Approximately 300 cc of blood loss was noted, with no other complications. Post-thrombectomy PA pressure was 30/12 mmHg (mPAP: 18 mmHg) and PA O_2_ saturation was 54%. The patient was discharged on Eliquis with an O_2_ saturation of 97% on room air.

## Discussion

Although better outcomes have been associated with the use of systemic thrombolytics, the risk of emerging intracranial hemorrhage has significantly increased^5^. The clinical implications of local catheter-directed thrombolysis infusion, together with catheter-directed mechanical thrombectomy have expanded considerably over the past years, owing to promising outcomes with a substantially decreased risk of intracranial bleeding compared to systemic thrombolysis^7,8^. This was evidenced by the FlowTriever Pulmonary Embolectomy Clinical Study (FLARE) achieving thrombectomy via the FlowTriever system in patients presenting with submassive PE patients; the major bleeding risk was 0.9% coupled with no reported events of intracranial hemorrhage. Moreover, FLARE depicted a significant 48 h reduction in RV/LV ratio (1.53–1.15, *P* < 0.0001): an independent predictor of mortality from PE. In addition, the average post-procedural mPAP decreased significantly to 27.8 mmHg from preprocedural pressure of 29,8 mmHg (*P* < 0.0001)^9^. In our case series, patients had a considerable decrease in mPAP after the procedure, especially when performed early, which reflects the association of early thrombectomy with optimal pulmonary hypertension treatment in cases of submassive PE.

The FlowTriever catheter system consists of a large aspiration guide catheter inserted into the PA, through which the FlowTriever device is advanced. Three self-expanding nitinol disks are unsheathed on the FlowTriever device to engage, disrupt, and extract the thrombus while aspirating and removing it through a 20 French guiding catheter^10^. In addition to PE, an Inari Flowtriver Catheter (Inari Medical Inc., Irvine, CA, USA) was used for other indications, as shown in (Table 1). This case series reflects our institution’s favorable early experience with the use of Inari’s novel FlowTriever large-bore mechanical thrombectomy device for the management of submassive PE.

**Table 1:** Overview of cases reporting the use of INARI FlowTriever mechanical thrombectomy in different clinical implications.

Case number	Age/Gender	Indications	Outcomes	Complications	References
1	66/F	DVT extending from the left common iliac vein to the left popliteal vein	Patent left lower extremity venous system	N/A	[11]
2	63/F	Phlegmasia Cerulea Dolens in the left lower extremity	immediate clinical resolution, including restoration of pedal pulses 45 min after thrombectomy	Minimal bleeding that resolved with manual compression	[12]
3	57/M	Right upper extremity deep venous thrombosis extending from the brachial vein to the subclavian vein	More than 90% reduction of the thrombus burden after a single pass	N/A	[13]
4	49/F	IVC thrombosis	Complete removal of the thrombus with no recurrent thrombus	N/A	[14]
5	53/M	Acute left ileo-caval DVT	Minimal remaining thrombus, complete resolution of the symptoms except mild left leg swelling with long periods of standing	N/A	[15]
6	29/F	phlegmasia cerulea dolens in the right lower extremity	Extensive thrombus burden removal after 4 passes	N/A	[16]
7	49/F	Left upper extremity DVT and central venous catheter malfunction	Minimal Clot burden with >90% patency of the brachial, axillary, and subclavian veins	Mild contrast extravasation in the proximal axillary vein treated with prolonged low-pressure balloon inflation	[17]
8	91/F	phlegmasia cerulea dolens in the left lower extremity	Nearly complete thrombus removal	N/A	[18]
9	55/F	PE in a patient on veno-arterial extracorporeal membrane oxygenation (VA-ECMO) associated with acute right heart failure	Extraction of several clots bilaterally and immediate improvement in the right ventricular function and dimension	Minimal blood loss	[19]
10	56/M	Aortic mural thrombus	Complete thrombus removal	N/A	[20]
11	68/M	Aortic and left femoropopliteal thrombi	Complete thrombi resolution	N/A	[20]
12	62/F	Left femoral and iliac DVT	Complete resolution of the occlusion after 3 passes	N/A	[21]
13	51/F	Massive PE with acute right heart failure	Removal of most of the thrombus and reestablishment of the blood flow	Persistent right ventricular failure that was managed with right ventricular mechanical support	[22]
14	75/M	sub massive PE	Removal of most of thr thrombus burden after 4 passes	N/A	[23]
15	50/M	Left proximal LE DVT	Complete removal of the thrombus with home discharge after 1 day	N/A	[24]
16	88/F	Massive PE and right atrial thrombus	no residual pulmonary clot and substantially reduced right heart thrombus burden	N/A	[25]
17	37/M	Bilateral submassive PE	Near Complete restoration of the blood flow, symptomatic recovery, and post-procedural mPAP decrease to 30 mmHg from 50 mmHg pre-procedure	Minimal blood loss	Case 1 (current)
18	79/M	Bilateral submassive PE	Near Complete restoration of the blood flow, symptomatic recovery, and post-procedural mPAP decrease to 30 mmHg from 32 mmHg pre-procedure	N/A	Case 2 (current)
19	48/F	Bilateral submissive PE	Complete restoration of the blood flow, symptomatic recovery, and post-procedural mPAP decrease to 18 mmHg from 30 mmHg pre-procedure	Minimal blood loss	Case 3 (current)

To date, there are no definitive guidelines regarding the use of mechanical-based thrombectomy in the management of submassive PE. The European Society of Cardiology/European Respiratory Society (ESC/ERS) recommends catheter-based thrombectomy in patients with high-risk PE, if thrombolysis fails or is contraindicated. Catheter-directed treatment in combination with ECMO may be considered if refractory circulatory collapse or cardiac arrest happens^26^. The American Heart Association (AHA)’s recommendation is dependent on the severity of the PE. In high-risk PE, the AHA recommends the use of interventional devices in cases of rapidly deteriorating hemodynamics to prevent mortality. However, in cases with intermediate-risk PE, the use of such devices is rationalized to hasten symptomatic recovery and prevent possible hemodynamic collapse resulting from progressive right-sided heart dysfunction. Nevertheless, no study has compared the long-term mortality benefits of large-bore mechanical thrombectomy devices in any subtype of PE^2^.

### What we have learned?

• Percutaneous mechanical thrombectomy utilizing the FlowTriever system is an effective and safe option for acute intermediate-high-risk pulmonary embolism associated with a significant improvement in the RV/LV ratio and minimal major bleeding.
• The FlowTriever system is an effective option for mechanical thrombectomy in cases of pulmonary embolism with concomitant clots in transit.
• Early utilization of FlowTriever in pulmonary embolism can achieve rapid normalization of pulmonary artery pressure and SpO2.
• Mechanical thrombectomy using FlowTriever is a safer option for clot removal in patients with contraindications to thrombolysis.
• Further research is needed to identify the long-term clinical benefits of mechanical thrombectomy.

### Conclusion

According to our experience in this case series, the use of the FlowTriever device for mechanical thrombectomy in patients with intermediate-risk PE improved patient outcomes. Early intervention leads to a significant decrease in mPAP and the resolution of patient symptoms. The device was also shown to be safe with minimal bleeding complications. Our case series complement the results of the FLARE study, which demonstrated the safety and efficacy of the INARI FlowTriever device. Because of its promising results, it is necessary to conduct more research studies on the FlowTriver device to compare its effectiveness and long-term mortality benefits to anticoagulants and systemic thrombolytic therapy in this set of patients and adjust management guidelines accordingly. Moreover, the role of large-bore catheter-based mechanical thrombectomy devices in managing acute pulmonary embolism and long-term potential in preventing chronic thromboembolic pulmonary hypertension (CTEPH) needs to be further investigated.
